# An siRNA Screen in Pancreatic Beta Cells Reveals a Role for *Gpr27* in Insulin Production

**DOI:** 10.1371/journal.pgen.1002449

**Published:** 2012-01-12

**Authors:** Gregory M. Ku, Zachary Pappalardo, Chun Chieh Luo, Michael S. German, Michael T. McManus

**Affiliations:** 1 Diabetes Center, University of California San Francisco, San Francisco, California, United States of America; 2 Division of Endocrinology, Metabolism, and Diabetes, Department of Medicine, University of California San Francisco, San Francisco, California, United States of America; 3 Department of Microbiology and Immunology, University of California San Francisco, San Francisco, California, United States of America; The University of North Carolina at Chapel Hill, United States of America

## Abstract

The prevalence of type 2 diabetes in the United States is projected to double or triple by 2050. We reasoned that the genes that modulate insulin production might be new targets for diabetes therapeutics. Therefore, we developed an siRNA screening system to identify genes important for the activity of the insulin promoter in beta cells. We created a subclone of the MIN6 mouse pancreatic beta cell line that expresses destabilized GFP under the control of a 362 base pair fragment of the human insulin promoter and the mCherry red fluorescent protein under the control of the constitutively active rous sarcoma virus promoter. The ratio of the GFP to mCherry fluorescence of a cell indicates its insulin promoter activity. As G protein coupled receptors (GPCRs) have emerged as novel targets for diabetes therapies, we used this cell line to screen an siRNA library targeting all known mouse GPCRs. We identified several known GPCR regulators of insulin secretion as regulators of the insulin promoter. One of the top positive regulators was *Gpr27*, an orphan GPCR with no known role in beta cell function. We show that knockdown of *Gpr27* reduces endogenous mouse insulin promoter activity and glucose stimulated insulin secretion. Furthermore, we show that *Pdx1* is important for *Gpr27*'s effect on the insulin promoter and insulin secretion. Finally, the over-expression of *Gpr27* in 293T cells increases inositol phosphate levels, while knockdown of *Gpr27* in MIN6 cells reduces inositol phosphate levels, suggesting this orphan GPCR might couple to Gq/11. In summary, we demonstrate a MIN6-based siRNA screening system that allows rapid identification of novel positive and negative regulators of the insulin promoter. Using this system, we identify *Gpr27* as a positive regulator of insulin production.

## Author Summary

Pancreatic beta cells are the only physiologic source of insulin. When these cells are destroyed in type 1 diabetics, there is uncontrolled hyperglycemia from complete insulin deficiency. In type 2 diabetes, these same cells fail to increase insulin secretion to compensate for peripheral insulin resistance leading to relative insulin deficiency. We constructed a novel screening system to find new regulators of insulin production in this critical cell type. Here, we describe a screen of the G protein coupled receptors (GPCRs) and show a role for orphan GPCR, *Gpr27*, in insulin promoter activity and insulin secretion. We propose that *Gpr27* is a novel target for diabetes therapeutics.

## Introduction

Nearly 13% of American adults have diabetes and these numbers continue to rise, mostly from an increase in type 2 diabetes [1], [2]. Although insulin resistance is a cardinal feature of type 2 diabetes, most people with insulin resistance do not develop diabetes because their pancreatic beta cells are able to compensate by increasing insulin production. However, if insulin production cannot match the increased demand imposed by insulin resistance, hyperglycemia and frank diabetes ensues. Over time, beta cell function further declines in most people with type 2 diabetes, resulting in the eventual failure of oral medications and the necessity of insulin therapy [3].

Improving insulin production and beta cell function is therefore a universal goal of diabetes therapeutics. We reasoned that an unbiased search for regulators of insulin production might reveal new diabetes drug targets. Therefore, we constructed a novel screening system to screen for genes important for insulin promoter activity. By screening siRNAs targeting all GPCRs, we identify several GPCRs that regulate insulin promoter activity and specifically characterize *Gpr27* as a novel regulator of insulin production.

## Results

### Generation of an insulin promoter reporter beta cell line

**Figure 1:**
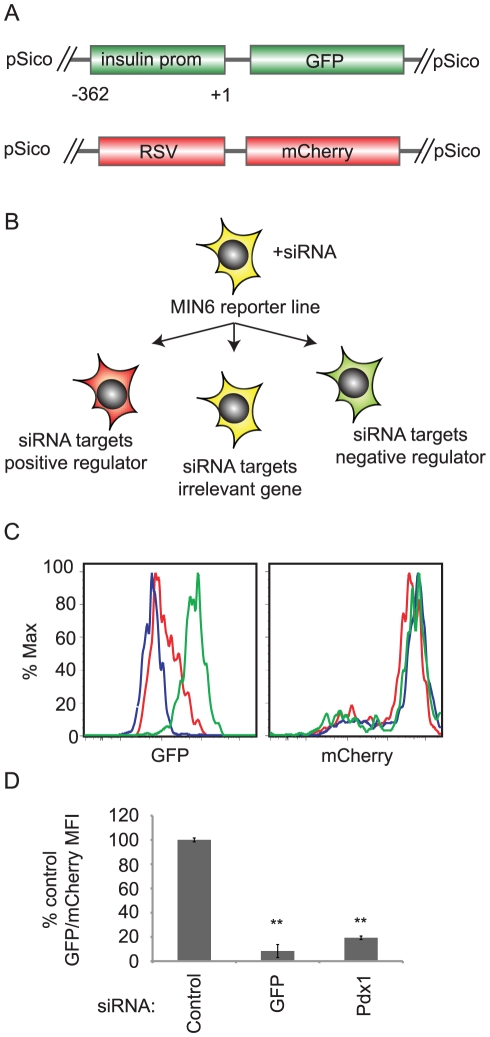
siRNA screening system to identify regulators of insulin promoter activity. A. Schema of reporter constructs integrated into the screening MIN6 cell line. These are pSico lentiviruses containing the proximal 362 bases of the human insulin promoter driving destabilized GFP and the rous sarcoma virus (RSV) promoter driving mCherry. B. After transfection with siRNAs, GFP and mCherry mean fluorescence intensity (MFI) are measured by flow cytometry. If the siRNA targets a positive regulator, GFP/mCherry MFI decreases. If the siRNA targets a negative regulator, the GFP/mCherry MFI increases. C. Control (green), Pdx1(red), or GFP(blue) siRNAs were transfected into the MIN6 reporter cell line. After 5 days, GFP (left panel) and mCherry (right panel) fluorescence were measured by flow cytometry. D. As in C. The ratio of GFP geometric mean fluorescence intensity (MFI) to mCherry MFI was calculated for each sample. The ratio was normalized to that of control siRNA transfected cells. Data shown are averages and standard error (n = 3). ** p<0.01 versus control siRNA.

To allow rapid evaluation of insulin promoter activity, the MIN6 mouse beta cell line was infected with a lentivirus that stably expresses destabilized GFP under the control of the proximal 362 base pairs of the human insulin promoter (Figure 1A) [4]. This insulin promoter fragment maintains a substantial proportion of promoter activity and tissue specificity while being compact enough to allow lentiviral delivery [5].

To favor single copy integration, the construct was delivered at a low multiplicity of infection (MOI) and a clonal line was selected. To generate an internal control reporter, the GFP positive subline was subsequently infected at a low MOI with a second lentivirus containing mCherry under the control of the constitutive rous sarcoma virus promoter (RSV) (Figure 1A). A stable clone expressing both constructs was isolated. In these cells, the ratio of GFP to mCherry fluorescence indicates human insulin promoter activity.

When transfected into this reporter line, siRNAs targeting activators of insulin gene transcription would be expected to reduce insulin promoter activity and reduce the GFP/mCherry ratio, while siRNAs targeting negative regulators of the insulin promoter should increase the GFP/mCherry ratio (Figure 1B). Indeed, transfection of an siRNA targeting the insulin gene transcription factor *Pdx1* reduced the GFP/mCherry ratio by 80% as compared to a non-targeting siRNAs (Figure 1C and 1D) [6].

### siRNA screen for non-odorant GPCR regulators of the insulin promoter

An RNAi library containing four independent siRNAs targeting the mouse GPCR-ome and selected GPCR related genes was transfected into the reporter cell line. The ratio of the GFP to mCherry fluorescence five days after transfection was calculated for each siRNA and the data were then analyzed using the redundant siRNA analysis (RSA) software [7]. To avoid off-target effects, each siRNA was transfected separately and only genes with more than one siRNA hit were selected for further analysis.

**Figure 2:**
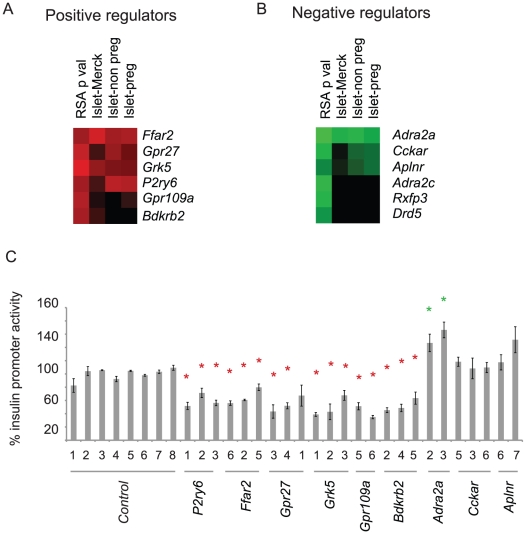
siRNA screen hit selection and initial confirmation. A. Heat map showing the top six putative positive regulators of the insulin promoter clustered based on RSA determined p value, and the base 10 log of the fragments per kilobase of exon model per million mapped reads (FPKM) value from three independent mRNA-seq data sets. B. As in B, but analyzed for negative regulators of insulin promoter activity. Islet-Merck refers to SRA008619 submitted by Merck, Islet-non preg refers to islets from non-pregnant mice, and Islet-preg refers to islets from pregnant mice [8]. C. Confirmation of hits from A and B. The indicated siRNAs were transfected into the screening cell line. GFP and mCherry was measured by flow cytometry. Error bars show standard error of three biological replicates performed (n = 3). * indicates p<0.05 versus >6 of the negative control siRNAs.

The top genes judged by RSA were then ranked by unsupervised clustering of each gene's RSA p value and its expression level in primary mouse islets, since only those genes expressed in primary cells are of biological interest (Figure 2A). Two publically available mouse islet mRNA-Seq data sets were used. One of these data sets has been previously published and consists of approximately four million mapped reads from islets isolated from female non-pregnant mice and approximately four million mapped reads from islets isolated from pregnant mice [8]. The second, submitted to the NCBI Short Read Archive by Merck, contains approximately 120 million reads from mouse islets (see methods). Because of these modest read numbers, some low abundance transcripts may be erroneously reported as being not expressed using this analysis [9].

siRNAs to the top six genes (*Ffar2, Gpr27, Grk5, p2ry6, Gpr109a, Bdkrb2*) that reduced insulin promoter activity and had detectable expression in primary mouse islets were transfected into the screening cell line for confirmation (Figure 2B). All six genes had at least 2 siRNAs confirm. For the siRNAs that increased GFP/mCherry, we retested the top three genes with high RSA scores and detectable expression in mouse primary islets – *Adra2a, Cckar, and Aplnr*. Of these three, only the known negative regulator of insulin secretion, *Adra2a*, confirmed with two independent siRNAs (Figure 2C).

Several of the positive regulators of the insulin promoter we identified were already known to stimulate insulin release in beta cells. Of particular interest was the orphan GPCR, *Gpr27*, which had no known role in insulin production but was previously found to be enriched in the mouse and human pancreatic islet [10], [11]. We subsequently tested all four siRNAs targeting *Gpr27* in the library set on an independently generated MIN6 reporter line expressing stable GFP under the control of the insulin promoter and mCherry under the control of the RSV promoter. All four siRNAs reduced GFP/mCherry fluorescence (Figure S1A). Furthermore, all 4 siRNAs efficiently reduced expression of the *Gpr27* mRNA (Figure S1B). We also confirmed that *Gpr27* is enriched in beta cell lines (beta TC and MIN6) compared to an alpha cell line (alpha TC) (Figure S2A) and is expressed in primary mouse beta cells (Figure S2B).

### Knockdown of *Gpr27* reduces the activity of the endogenous mouse insulin promoter in cultured cells and primary islets

**Figure 3:**
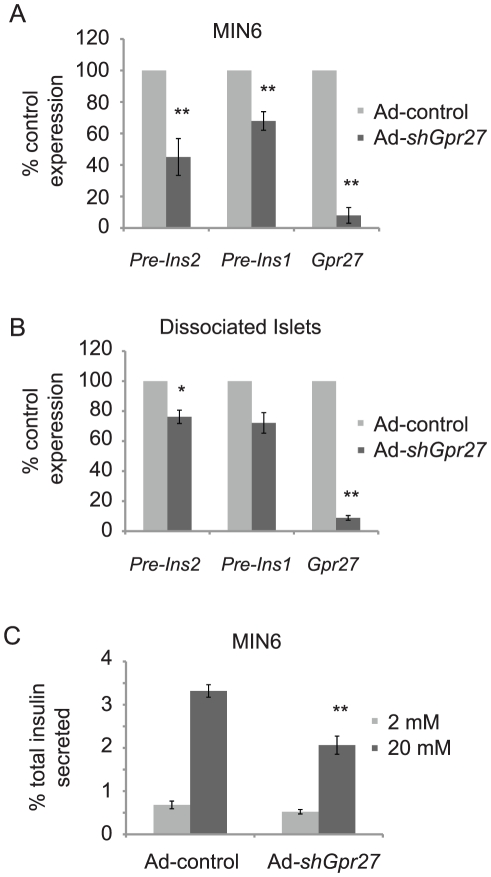
*Gpr27* is required for mouse insulin promoter activity and glucose stimulated insulin secretion. A. MIN6 cells were infected with either Ad-control or Ad-sh*Gpr27*. Three days after infection, RT-qPCR was performed for the indicated genes. The data are plotted as % expression compared to control adenovirus with standard error (n = 3). B. As in A, but dispersed primary mouse islets were infected with Ad-control or Ad-sh*Gpr27*. After 3 days, infected cells were sorted by flow cytometry of GFP positive cells and RT-qPCR was performed for the indicated genes (n = 3). C. MIN6 cells were infected with either Ad-control or Ad-sh*Gpr27*. Three days after infection, glucose stimulated insulin secretion was measured by ELISA after 1 hour of static incubation at either 2 mM or 20 mM glucose. Data are represented as the average of fractional insulin secretion with standard error (n = 9). *p<0.05, **p<0.005 versus Ad-control.

Since the screen was based on a human insulin promoter fragment, we measured the effect of *Gpr27* knockdown on the endogenous mouse *Ins2* and *Ins1* genes. Because mature insulin mRNAs have a half-life of nearly 80 hours, we measured insulin pre-mRNAs as previously described [6], [12]. MIN6 cells infected with a *Gpr27* shRNA expressing adenovirus (Ad-shGpr27) had a 40–60% reduction in pre-ins2 and pre-ins1 levels compared to control adenovirus (Ad-control) (Figure 3A). To confirm these findings in primary beta cells, we infected intact primary mouse islets with these same adenoviruses. At a high MOI, we were only able to obtain 50% infection rates as measured by flow cytometry, presumably reflecting poor adenovirus penetration into the core of the mouse islet [13]. Therefore, intact islets were dissociated prior to adenovirus infection. Three days after infection, cells were isolated by flow cytometric sorting for GFP and RT-qPCR was performed. Knockdown of *Gpr27* produced a significant ∼30% reduction of pre-ins2 (p = 0.03). Concomitantly, there was a nearly significant 30% reduction in the less abundant pre-ins1 message (p = 0.055) (Figure 3B).

### Knockdown of *Gpr27* impairs glucose stimulated insulin secretion

While insulin production requires insulin promoter activity, minute-to-minute changes in plasma insulin levels are controlled by insulin secretion. Therefore, we asked if *Gpr27* knockdown would affect glucose stimulated insulin secretion. Infection of MIN6 cells with Ad-control at an MOI necessary to get >90% infection inhibited glucose stimulated insulin secretion (data not shown). Therefore, we infected MIN6 cells at a lower MOI to achieve approximately 60% infection and measured glucose stimulated insulin secretion from this mixed population by batch incubation. Ad-shGpr27 infected MIN6 cells secreted ∼40% less insulin at 20 mM glucose compared to Ad-control infected cells (Figure 3C). There was no statistically significant difference at 2 mM glucose. Notably, we did not detect a difference in total insulin as normalized to total protein concentration (Ad-control = 27.9+/−1.1 mg insulin per g of total protein; Ad-shGpr27 = 29.4+/−0.94 mg insulin per g of total protein, p value = 0.13). This was not unexpected since the half-life of insulin mRNA is ∼80 hours and the knockdown of *Gpr27* was limited to 72 hours due to adenovirus toxicity after that time point. We conclude that *Gpr27* plays a measurable role in insulin secretion in addition to insulin promoter activity.

### *Gpr27's* effect on the insulin promoter and insulin secretion requires *Pdx1*

**Figure 4:**
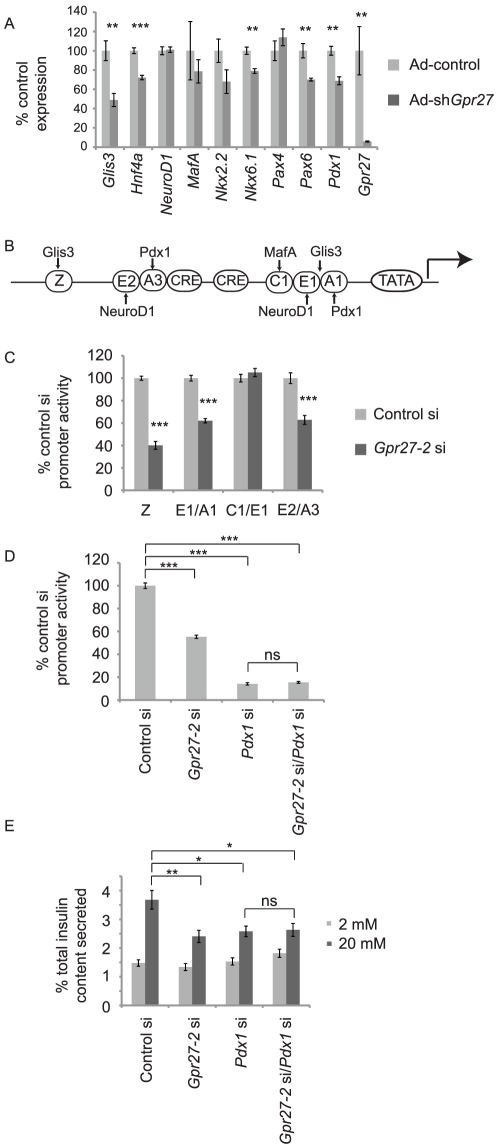
*Gpr27* knockdown affects multiple transcription factors and requires *Pdx1* for its effect on the insulin promoter. A. MIN6 cells were infected with Ad-control or Ad-sh*Gpr27*. Three days after infection RT-qPCR was performed for the indicated genes. Expression level normalized to that of Ad-control are plotted with standard error (n = 6). B. Schematic of the human insulin promoter with selected regulatory sequences and transcription factors that bind to these elements. C. MIN6 cells were cotransfected with the indicated siRNA, insulin promoter firely luciferase construct and thymidine kinase renilla luciferase construct. Two days after transfection, firefly and renilla luciferase activity were measured. The ratio of firefly to renilla luciferase was normalized to the control siRNA. Average and standard error are plotted (n = 6–9) D. As in C using human insulin promoter −362 firefly luciferase (n = 6–9). E. MIN6 cells were transfected with the indicated siRNAs and glucose stimulated insulin secretion was measured after 5 days. Fractional insulin secretion is shown (n = 12). * p<0.05 ** p<0.005 *** p<0.0005 versus control siRNA or control adenovirus (at high glucose part E).

To define the mechanism of *Gpr27* action, we measured transcript levels of selected regulators of the insulin promoter by RT-QPCR in MIN6 cells after Ad-shGpr27 infection. *Glis3*, *Pax6*, *Nkx6.1*, *HNF4a*, and *Pdx1* were reduced after *Gpr27* knockdown while others including *MafA*, *NeuroD1*, and *Pax4* were unchanged (Figure 4A). Concordant with this expression data, *Gpr27* knockdown reduced the transcriptional activity of mini-enhancers that bind to *Glis3* and *Pdx1* (Z, E1/A1, E2/A3) while *Gpr27* knockdown had no effect on mini-enhancers that bind to *MafA* and *NeuroD1* (C1/E1) (Figure 4B and 4C).

Since *Pdx1* is required for insulin promoter activity and insulin secretion [14], [15], we asked if *Pdx1* is required for the effect of *Gpr27's* on the insulin promoter. By luciferase assay, we found that the single knockdown of *Pdx1* reduced insulin promoter activity by 90% and *Gpr27* knockdown alone reduced insulin promoter activity by 40%. However, the knockdown of both *Gpr27* and *Pdx1* had no additional effect over the single knockdown of *Pdx1*, showing that *Pdx1* is important for the effect of *Gpr27* on the insulin promoter (Figure 4D). Importantly, double knockdown of both *Gpr27* and *Pdx1* was as efficient as single knockdown (Figure S3).

We then asked if *Pdx1* was required for the effect of *Gpr27* on insulin secretion. The knockdown of *Pdx1* reduced fractional insulin secretion at 20 mM glucose and total insulin content (Figure 4E and Figure S4). As with the adenoviral knockdown of *Gpr27*, an siRNA to *Gpr27* reduced glucose stimulated insulin secretion. However, the knockdown of *Gpr27* in addition to *Pdx1* did not further reduce insulin secretion at 20 mM glucose. We conclude that *Gpr27* plays a measurable role in insulin secretion and insulin promoter activity via a mechanism involving *Pdx1*.

### *Gpr27* increases IP1 levels

**Figure 5:**
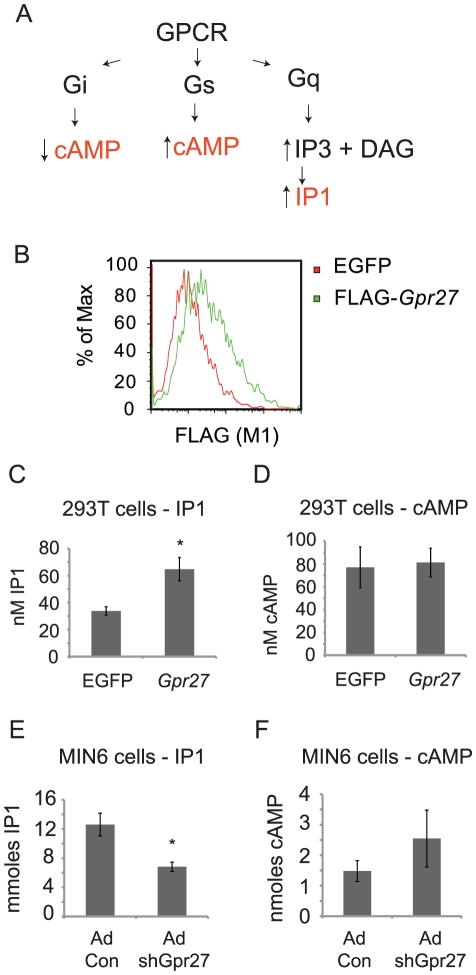
*Gpr27* positively regulates inositol phosphate levels. A. Schema of canonical GPCR signaling pathways and resulting expected changes in second messengers cAMP and IP3. B. HEK293T cells were transiently transfected with either GFP plasmid (control) or FLAG-*Gpr27* plasmid. 24 hours after transfection cells were analyzed by flow cytometry for extracellular FLAG. C. As in B, but cells were lysed and assayed for IP1. D. As in C, but lysates were analyzed for cAMP. E. MIN6 cells were infected with control or *Gpr27* knockdown adenovirus and 3 days later, cells were lysed and IP1 was measured. F. As in E, but cAMP was measured. For C–F, average and standard error is plotted (n = 9); * p<0.005 versus control.

G protein coupling software analysis predicts that *Gpr27* could function via Gi or Gq/11 signaling pathways [16]. Since *Gpr27* is already expressed in MIN6 cells, we ectopically expressed mouse *Gpr27* in HEK293T cells. Robust expression of FLAG-tagged *Gpr27* was detected by 24 hours on the surface of the majority of cells (Figure 5B). We then measured cAMP and IP1 – higher cAMP would indicate Gs coupling, lower cAMP would indicate Gi coupling and higher IP1 would indicate Gq/11 coupling (Figure 5A). *Gpr27* expression resulted in a 2-fold elevation of IP1 levels while leaving cAMP levels unchanged (Figure 5C and 5D) showing that in this heterologous cell type, *Gpr27* may activate the Gq/11 pathway.

If *Gpr27* activates Gq/11 in beta cells, then IP1 levels should be reduced in MIN6 cells after knockdown of *Gpr27*. Therefore, we measured IP1 levels and cAMP levels in MIN6 cells after *Gpr27* knockdown. Indeed, knockdown of *Gpr27* resulted in reduced IP1 levels while cAMP levels were not significantly changed (Figure 5E and 5F). Taken together, these data show that *Gpr27* positively regulates inositol phosphate levels, supporting a role for *Gpr27* in activating the Gq/11 pathway.

## Discussion

To identify new regulators of the insulin promoter, we developed a novel siRNA screening system in MIN6 cells that allows rapid measurement of insulin promoter activity. As an initial test of the system, an siRNA screen of the GPCR-ome was performed. The RSA algorithm was used to select hits in order to capitalize on the four fold redundancy of the siRNA library [7]. To further increase the specificity of the screen, at least 2 siRNAs must have been identified for a gene to be a hit. The top RSA hits were then prioritized by expression level in mouse primary islets. Besides filtering out genes expressed in MIN6 but not in primary islets, this step also eliminates off-target hits. On the other hand, hit genes with low expression may have been erroneously eliminated because they were below the limit of detection of the mRNA-seq data available at this time [17]. Nonetheless, this filtering step allowed us to focus on genes with reasonable expression in primary cells.

While the confirmation rate for siRNAs to positive regulators was 100%, the confirmation rate for negative regulators was only 33%. This is likely due, in part, to the more modest effect of these siRNAs (∼20–30% increase in GFP/mCherry ratio) as compared to the reconfirmed *Adra2a*(∼50%), a known negative regulator of insulin secretion.

We identified several other known regulators of insulin secretion as regulators of the insulin promoter. The bradykinin receptor 2 mediates increases in insulin secretion in beta cells [18], [19]. Pyrimidinergic receptor 6 (*p2yr6*) agonists augment insulin release and this receptor participates in an autocrine feedback loop that potentiates insulin secretion [20], [21]. The free fatty acid receptor 2, which has been hypothesized to play a role in beta cells, was also identified as a positive regulator of the insulin promoter in our screen [22]. Several other receptors were identified in the screen that have no known role in beta cells and these may merit further investigation. Of note, *Glp1r* was not identified in this screen for a trivial reason; siRNAs targeting this gene were not included in the commercial screening set. Given the nature of our screen, hits would be predicted to either have basal activity or have ligand present in the culture conditions as has been described for *p2yr6* [21].

We were most intrigued by the orphan GPCR, *Gpr27* [23]. Previous studies have shown that it is enriched in the pancreatic islets of both human and mouse [10], [11]. Detailed mouse tissue profiling of *Gpr27* expression by RT-QPCR shows high expression in the mouse brain with lower expression in the islet and heart [24]. Furthermore, *Gpr27* mRNA is up-regulated in Neurogenin3 positive endocrine precursors in the developing mouse pancreas [11]. Conversely, *Gpr27* is 8-fold down regulated in the Neurogenin3 knockout pancreas [25], [26]. Taken together, these data suggest *Gpr27* is an endocrine pancreas specific gene.

We confirmed that knockdown of *Gpr27* reduces the activity of human insulin promoter reporters, levels of endogenous mouse *Ins2* pre-mRNA, and glucose stimulated insulin secretion. Importantly, *Gpr27* knockdown also reduces the levels of endogenous *Ins2* pre-mRNA in dissociated primary mouse islets. We also found that the mRNAs for multiple transcription factors that activate the insulin promoter (*Glis3, Pdx1, HNF4a*) were reduced by *Gpr27* knockdown. Other transcription factors critical for beta cell development were also reduced including *Nkx6.1* and *Pax6*. In agreement with the reduction in their expression, only *Pdx1* and *Glis3* binding mini-enhancers were affected by *Gpr27* knockdown (Figure 4C). Finally, there was no further reduction in insulin promoter activity when adding *Gpr27* knockdown to *Pdx1* knockdown. A limitation of this double knockdown experiment is that given the very strong effect of *Pdx1* knockdown alone on insulin promoter activity, a further reduction with *Gpr27/Pdx1* double knockdown may be either below our limit of detection or simply reflect no remaining insulin promoter activity.

How might *Gpr27* affect both insulin transcription and glucose stimulated insulin secretion? The Gq/11 pathway was an obvious candidate as the expression of *Gpr27* in HEK 293T cells increased IP1 levels while the knockdown of *Gpr27* reduced IP1 levels in MIN6 cells. Furthermore, triggering of an engineered Gq/11-coupled GPCR in beta cells increases steady state insulin mRNA levels and insulin secretion [27]. However, *Pdx1* levels did not change after triggering this Gq/11-coupled GPCR [27] and Gq/11 knockout beta cells have normal levels of *Ins1* and beta cell transcription factor mRNAs [21]. Therefore, even if *Gpr27* directly couples to Gq/11, *Gpr27* may affect insulin secretion and insulin promoter activity independent of Gq/11 as has recently been demonstrated for the M3 receptor [28].

Another candidate for mediating the effects of *Gpr27* on insulin promoter and insulin secretion was *Pdx1* since it is known to positively regulate both insulin transcription and insulin secretion [14], [15]. We found that the double siRNA knockdown of *Gpr27* and *Pdx1* produced no further reduction in insulin secretion over *Pdx1* knockdown alone, suggesting that *Pdx1* is important for *Gpr27's* effect on insulin secretion. In combination with the reduction in *Pdx1* mRNA by Gpr27 knockdown, these data suggest Gpr27 functions upstream of *Pdx1*. However, the double knockdown data do not exclude the possibility that a *Pdx1* lies in a parallel pathway to *Gpr27* and these two pathways intersect upstream of insulin secretion.

Taken together, these data suggest that a linear pathway connecting *Gpr27* to a single G protein and a single regulatory element in the insulin promoter is overly simplistic. Indeed, a single GPCR can trigger multiple G proteins (reviewed by [29]), can trigger a combination of G protein dependent and independent pathways [30], and can function as heterodimers [31]. Likewise, the insulin promoter contains multiple elements that are both redundant and cooperative [32]. The complexity of these systems highlights the advantage of using a broad, unbiased approach to finding new and unexpected regulators of the insulin promoter. Here, we used such a system to identify a novel GPCR regulator of both insulin secretion and insulin promoter activity – *Gpr27*. Based on its islet expression and its positive effects on the insulin promoter and insulin secretion, we suggest that *Gpr27* may be a novel target for diabetes therapies.

## Materials and Methods

### Cell culture

MIN6 cells were a gift from Dr. Miyazaki. Alpha TC and beta TC were a gift from Dr. Hanahan. Cells were maintained in high glucose DMEM with 10% fetal bovine serum, and 71.5 mM beta-mercaptoethanol. Sublines were isolated by limiting dilution. Original passage lines were used between passage 25–40. Sublines were used at passages 5–10.

### Promoter constructs

Human insulin promoter deletions have been previously described [5]. Promoters were subcloned from pFoxCAT into pFoxLuc [33]. For the lentiviral reporter, the human −362 promoter region was cloned upstream of destabilized GFP or GFP and this cassette was used to replace the U6/CMV-EGFP in pSicoR. pSicoR-RSV-mCherry was created by replacing the U6/CMV of pSicoR mCherry with the RSV promoter [5]. Mini-enhancer reporter constructs have also been previously described [5], [34]. They were subcloned upstream of a minimal thymidine kinase promoter-firefly luciferase reporter.

### siRNA transfection

Approximately 5,000 MIN6 cells were transfected in 96 well plates using HiPerfect (Qiagen) with a final siRNA concentration of 25 nM. Cells were analyzed by flow cytometry (LSRII, BD) 5 days after transfection and the geometric mean fluorescence intensity of GFP was normalized to that of mCherry. If the knockdown of GFP by an anti-GFP siRNA was not >80%, the transfection of that plate was considered to be a technical failure and the plate was discarded. This occurred on 1 out of 20 plates and for this reason some genes were only targeted by 3 siRNAs (including *Gpr27*). Each well was normalized to the negative control siRNA on that 96 well plate. For the confirmation assay for *Gpr27* siRNAs, a distinct MIN6 human insulin promoter-GFP/RSV-mCherry reporter line was transfected with the indicated siRNAs with Lipofectamine RNAiMax for 5 days and GFP and mCherry were measured.

### GPCR expression analysis and hierarchical clustering

Mouse islet mRNA-seq data was downloaded from the NCBI Short Read Archive (SRP000752 and SRP002569) and FPKM values were calculated using the TopHat and Cufflinks software using the NCBI RefSeq as the reference. Log FPKM and negative log RSA p values were clustered using Cluster 3.0 and heat maps were plotted with JavaTreeView.

### siRNA and qPCR probes

siRNAs were obtained from Qiagen. All custom Taqman probes had a confirmed PCR efficiency of between 95–110%. Samples without reverse transcriptase did not amplify. See Text S1 for sequences of custom probes. Taqman probes to mouse *Glis3, MafA, Pdx1, NeuroD1, Pax4, Nkx6.1, HNF4a* were obtained from Applied Biosystems. Negative control siRNAs for the reconfirmation assay were All-Stars Negative Control (1027280), Negative Control (1022076), Unspecific-Luciferase-1 (1022070), Unspecific-Luciferase-2 (1022073), Hs_LMNA_11 (1022050), Mm_Lmna_5 (SI02655450), Hs_GAPD_5 (SI0253266), Hs_ACTB_1 (1022168).

### RT–qPCR

Total RNA was isolated by Trizol (Invitrogen). The RNA was DNase I treated (Turbo DNase, Ambion) and reverse transcription was performed (Superscript III, Invitrogen) using a combination of random hexamers and oligo dT primers. For cell line experiments, each qPCR reaction used between 10–30 ng of total RNA equivalent. To convert to arbitrary linear units, the following formula was used: (2∧15)*(2∧(deltaCT to beta-glucuronidase).

### Isolation of MIP-GFP positive cells

Islets from 12–30 week old MIP-GFP mice were isolated by the UCSF Islet Production Core. Islets were digested with trypsin until single cell suspensions were obtained. Cells were sorted by flow cytometry (Aria II, BD or MoFlo, DakoCytomation) into GFP positive and negative fractions and total RNA was isolated. 20 ng of total RNA equivalent was loaded per QRT-PCR reaction.

### Luciferase assays

140,000 MIN6 cells were transiently transfected in 24 well plates with the relevant siRNA (5 pmoles), the indicated insulin reporter firefly luciferase plasmid (100 ng), and pRL-TK(Promega) (25 ng) using Lipofectamine 2000 (Invitrogen). For double siRNA knockdowns, 5 pmoles of each siRNA or 10 pmoles of control (anti-GFP) were used. Two days after transient transfection, firely and renilla luciferase were measured using the Dual Luciferase Assay (Promega).

### Gpr27 expression in HEK 293T

*Gpr27* was cloned by PCR from mouse genomic DNA downstream of a viral signal sequence and amino terminal FLAG epitope tag [35]. This cassette was used to replace the EGFP in pSicoR. HEK 293T cells were transiently transfected with either pSicoR-EGFP or pSicoR-FLAG-Gpr27 using LT1 (Mirus).

### FLAG flow cytometry

293T cells were dissociated with PBS without Ca or Mg, stained with M1 anti-FLAG antibody and a Goat anti-mouse secondary antibody coupled to Alexa-594 (Invitrogen).

### IP1 and cAMP assays

For 293T, one day after transient transfection in 24 well plates, cells were placed in stimulation buffer (HTRF) for 30 minutes at 37 degrees. The stimulation buffer was then removed and the cells were lysed using the kit lysis buffer. IP1 and cAMP were then measured as directed by the protocol in 384 well plates (HTRF). IP1 and cAMP levels were normalized to live cells numbers counted from duplicate wells. Viable cells counts from *Gpr27* transfection were within 20% of control plasmid transfection. For MIN6 cells, 125,000 cells were infected with *Gpr27* shRNA or control adenovirus at an MOI of 200 and grown in 24 well dishes (resulting in nearly ∼95% infection). Three days after infection, the cells were placed in stimulation buffer (HTRF) for 30 minutes at 37 degrees. The stimulation buffer was then removed and the cells were lysed in 1% Triton-X100, 50 mM HEPES pH 7.0, NaF 15 mM. The lysate was pre-cleared by centrifugation at 14,000 rpm for 10 minutes. A fraction of the lysate was taken for protein quantitation(micro-BCA, Pierce), IP1 or cAMP measurement (HTRF). Data were normalized to total protein content.

### shRNA adenovirus construction

The *Gpr27* shRNA was cloned into a modified version of pSicoR with a BstXI site replacing the HpaI site. The mouse U6 promoter and *Gpr27* shRNA were then subcloned from pSicoR and placed upstream of the CMV-GFP marker in pAdTrack [36], [37]. Adenovirus was prepared and tittered as previously described [38].

### Knockdown in primary mouse islets

Islets were isolated by the UCSF Islet Production Core Facility from 8–12 week old C57Bl/6 male mice. After 24 hours of culture in RPMI and 10% FBS, islets were trypsinized until single cell suspensions were obtained. The dissociated islet cells were resuspended in RPMI+10% FBS and infected with adenovirus at multiplicity of infection (MOI) of 25. Three days after infection, the cells were sorted by flow cytometry (Aria II, BD) for GFP positive cells (50–75% of the live population) and RT-qPCR was performed. The knockdown of pre-ins2, pre-ins1 or *Gpr27* from *Gpr27* shRNA adenovirus infected cells was calculated by the delta-delta CT method compared to the control adenovirus infection.

### Insulin secretion assays

For the adenovirus assays, approximately 500,000 MIN6 cells were infected with the indicated adenoviruses at an MOI of 100 in 6 cm dishes in complete media. Three days after infection, the infection rate was ∼60% by FACS for GFP. Cells were washed 5 times in KRBH buffer (10 mM HEPES pH 7.4, 130 mM NaCl, 5 mM KCl, 1.25 mM KH2PO4, 1.25 mM MgSO4, 2.68 mM CaCl2, 5.26 mM NaHCO3) with 2 mM glucose and rested for 2 hours at 37 degrees. Cells were then washed an additional 3 times with 2 mM glucose KRBH and incubated in 3 mL of 2 mM glucose KRBH for 1 hour at 37 degrees. This supernatant was collected and replaced with 20 mM glucose KRBH for 1 hour at 37 degrees. Cells were washed with PBS before lysis in 50 mM Tris-HCl pH 8.0, 150 mM NaCl, 1% Triton X-100 with protease inhibitors. Lysates were spun at 14,000 rpm for 10 minutes and supernatants were spun at 5000 rpm for 5 minutes before analysis by an Ultrasensitive Insulin ELISA (Mercodia). Total protein was measured by Micro-BCA (Pierce). Total insulin was normalized to total protein in the lysate. For the siRNA transfections, 20,000 MIN6 cells were transfected per well of a Corning CellBIND 96 well plate with 25 nM of each siRNA (or 50 nM of control siRNA) using Lipofectamine RNAiMax. 5 days after transfection, cells were washed in KRBH with 2 mM glucose twice, then incubated for 2 hours at 37 degrees, then washed again with KRBH 2 mM glucose twice, then incubated for one hour with KRBH 2 mM, then KRBH with 20 mM glucose for 1 hour. Lysates were prepared in 75 uL of lysis buffer as above. Due to the lower cell numbers in the 96 well plate assay, total insulin was normalized to total genomic DNA measured by Qubit High Sensitivity DNA kit (Life Technologies).

### Statistical analysis

For siRNA primary confirmation assay, an independent, two sample, one tailed t-test was used. For the primary islet adenovirus knockdown of *Gpr27* an independent, one sample, two tailed t-test was used. All other p values were calculated with an independent, two sample, two tailed t-test.

### Ethics statement

Animal experiments were approved by the UCSF Institutional Animal Care and Use Committee (Protocol AN082433-02) with care taken to avoid any unnecessary suffering. Animals were maintained in accordance with the applicable portions of the Animal Welfare act and the DHHS Guide for the Care and Use of Laboratory Animals.

## Supporting Information

Figure S1 Multiple *Gpr27* siRNAs potently knockdown *Gpr27* and reduce insulin promoter activity. A. The indicated siRNA was transfected into MIN6 insulin promoter-GFP, RSV-mCherry cells and five days after transfection, GFP and mCherry fluorescence were measured by flow cytometry. Data are normalized to the GFP/mCherry fluorescence of the control siRNA. Error bars show standard error (n = 3). B. As in A but RT-QPCR was performed for *Gpr27* (n = 4). * p<0.01.
(PDF)

Figure S2 *Gpr27* is enriched in beta cell lines and is expressed in primary beta cells. A. RT-qPCR was performed on the indicated cell types for *Gpr27*. B. RT-qPCR was performed on intact primary mouse islets or on GFP high and low cells from islets of insulin promoter GFP transgenic mice. Error bars show standard error. Data shown are from two independent islet isolations, dissocations and flow cytometric sortings performed on two different days.
(PDF)

Figure S3 Double siRNA knockdowns in result in efficient target knockdown. MIN6 cells were transfected with the indicated pairs of siRNAs. 5 days after transfection, total RNA was extracted and RT-QPCR for the indicated genes were performed. N = 3–6 biololgical replicates. * p<0.05. ** p<0.005.
(PDF)

Figure S4 Pdx1 knockdown reduces total insulin levels. MIN6 cells were transfected with the indicated siRNAs and glucose stimulated insulin secretion was measured after 5 days. Here, total insulin normalized to total genomic DNA content is presented. These data correspond to the data shown in Figure 4E. N = 12 biological replicates, *p<0.01.
(PDF)

Text S1 Sequences of QPCR probes, siRNAs, and shRNA used in this study.
(DOCX)
