# Supplementary figures and images for: An siRNA Screen in Pancreatic Beta Cells Reveals a Role for Gpr27 in Insulin Production
Source: PLoS Genet. 2012 Jan 12;8(1):e1002449. doi: 10.1371/journal.pgen.1002449 (PMC3257298; doi:10.1371/journal.pgen.1002449)

A

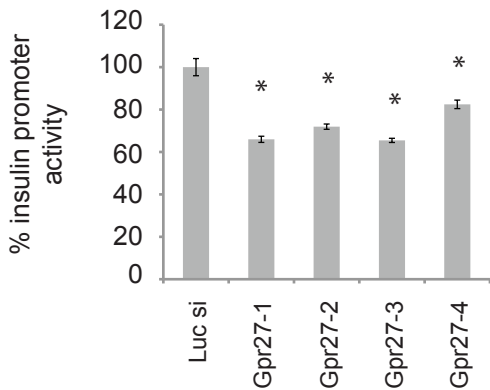

B

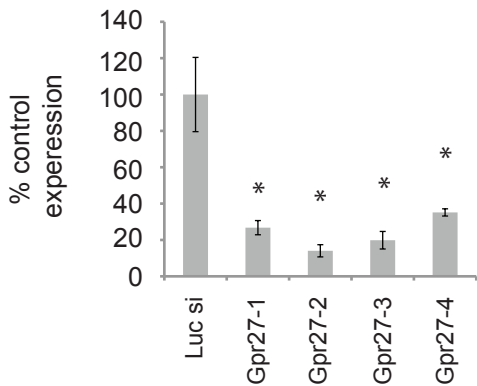

Supplement: Figure S1 — Multiple Gpr27 siRNAs potently knockdown Gpr27 and reduce insulin promoter activity. A. The indicated siRNA was transfected into MIN6 insulin promoter-GFP, RSV-mCherry cells and five days after transfection, GFP and mCherry fluorescence were measured by flow cytometry. Data are normalized to the GFP/mCherry fluorescence of the control siRNA. Error bars show standard error (n = 3). B. As in A but RT-QPCR was performed for Gpr27 (n = 4). * p<0.01. (PDF) [file pgen.1002449.s001.pdf]

A

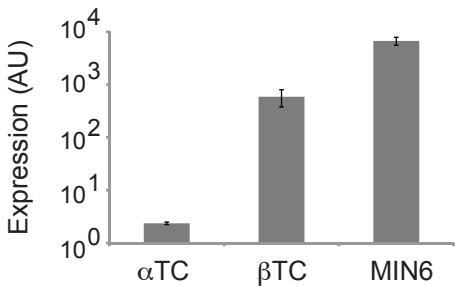

B

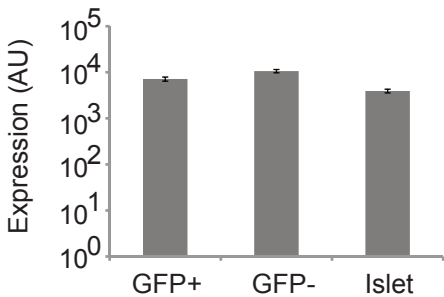

Supplement: Figure S2 — Gpr27 is enriched in beta cell lines and is expressed in primary beta cells. A. RT-qPCR was performed on the indicated cell types for Gpr27. B. RT-qPCR was performed on intact primary mouse islets or on GFP high and low cells from islets of insulin promoter GFP transgenic mice. Error bars show standard error. Data shown are from two independent islet isolations, dissocations and flow cytometric sortings performed on two different days. (PDF) [file pgen.1002449.s002.pdf]

A

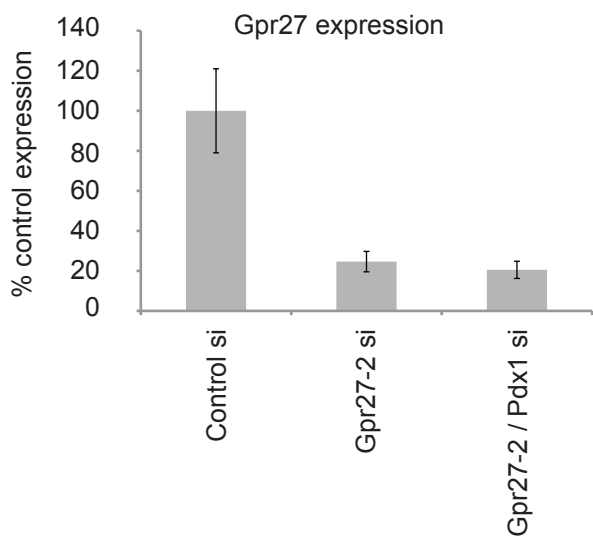

B

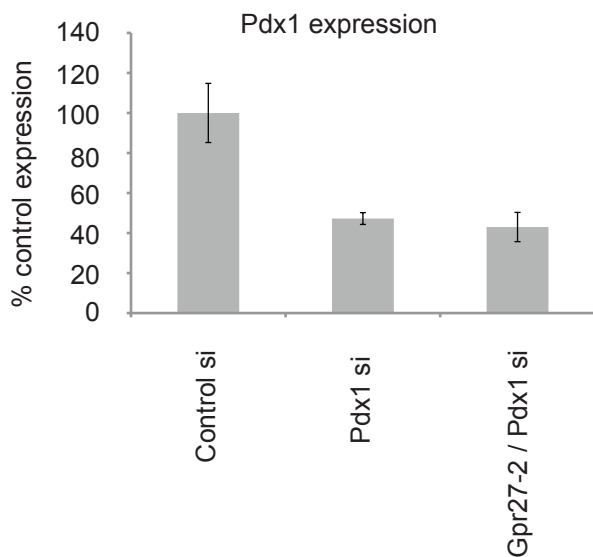

Supplement: Figure S3 — Double siRNA knockdowns in result in efficient target knockdown. MIN6 cells were transfected with the indicated pairs of siRNAs. 5 days after transfection, total RNA was extracted and RT-QPCR for the indicated genes were performed. N = 3–6 biololgical replicates. * p<0.05. ** p<0.005. (PDF) [file pgen.1002449.s003.pdf]

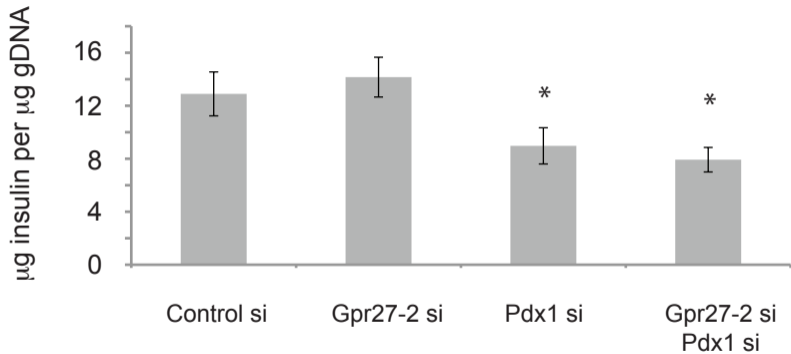

Supplement: Figure S4 — Pdx1 knockdown reduces total insulin levels. MIN6 cells were transfected with the indicated siRNAs and glucose stimulated insulin secretion was measured after 5 days. Here, total insulin normalized to total genomic DNA content is presented. These data correspond to the data shown in Figure 4E. N = 12 biological replicates, *p<0.01. (PDF) [file pgen.1002449.s004.pdf]
