# Supplementary material for: An siRNA Screen in Pancreatic Beta Cells Reveals a Role for Gpr27 in Insulin Production
Source: PLoS Genet. 2012 Jan 12;8(1):e1002449. doi: 10.1371/journal.pgen.1002449 (PMC3257298; doi:10.1371/journal.pgen.1002449)
Supplement: Text S1 — Sequences of QPCR probes, siRNAs, and shRNA used in this study. (DOCX) [file pgen.1002449.s005.docx]

| **QPCR Probes** |  |  |  |
| --- | --- | --- | --- |
|  | **Forward** | **Reverse** | **Probe** |
| Gpr27 | cctcggtgtggctgacatt | ggaaactgggctcggaaac | atcaaccccgtggtgtgtttcctcttc |
| Pre-ins1 | gggaccacaaagatgctgtt | tgggctcctctcttacatgg | ctgttggtgcacttcctacccctg |
| Pre-ins2 | agtggcagaactcaccttg | gggagcgtggcttcttc | cggcgggacatgggtgtgta |
| GUS | ctcatctggaatttcgccga | ggcgagtgaagatccccttc | cgaaccagtcaccgctgagagtaatcg |
| Pdx1 | gacccagagtgtggacgtg | tctccggctatacccaactg | cagctggataagggaacttaacctaggcgt |
|  |  |  |  |
| **siRNA** | **Target sequence** |  |  |
| GFP | cggcaagctgaccctgaagttcat |  |  |
| Gpr27_1 | GAGGCTGTGCAAGATGTTCTA | |  |
| Gpr27_2 | ctgaaaggcattggtttgtga |  |  |
| Gpr27_3 | CTGAAAGGCATTGGTTTGTGA | |  |
| Gpr27_4 | CCGGGAGCTGAGGGACTGTTT | |  |
| Pdx1 | cagctggataagggaacttaa |  |  |
| Luc | AACTTACGCTGAGTACTTCGA | |  |
| P2ry6_1 | CAGGATGTTGTGACAAGATAA | |  |
| P2ry6_2 | ATGGTTGAAGCTTTCTCTCAA | |  |
| P2ry6_3 | CAGATGATCAAGGATCTGCTA | |  |
| Gpr43_6 | AAGCATAGCTCCAGGATACAA | |  |
| Gpr43_2 | CACCATCGTCATCATCGTTCA | |  |
| Gpr43_5 | GACATTGACAATGACCTGAAA | |  |
| Gprk5_1 | CTGGATGATTATGGCCACATA | |  |
| Gprk5_2 | CAGGTTCGGGCCACTGGTAAA | |  |
| Gprk5_3 | ACCGAATAAATTCAAACCACA | |  |
| Gpr109b_5 | TTCAATAAATTTCGACTGAAA | |  |
| Gpr109b_6 | AACCGCTGCCTTCGAAAGAAA | |  |
| Bdkrb2_2 | CAGCGTGTTCTTCCTGCACAA | |  |
| Bdkrb2_4 | CATGATCTACATGAACCTGTA | |  |
| Bdkrb2_5 | CACCGCCTGCGTCATCGTCTA | |  |
| Adra2a_1 | CTGCAAGATCAACGACCAGAA | |  |
| Adra2a_3 | TAAGGTGTGGTGTGAGATCTA | |  |
| Cckar_5 | AAGGATGATCTCTACAGTATA | |  |
| Cckar_3 | CAAATGGATATTGGAACTCAA | |  |
| Cckar_6 | AAGCTTGAGGTTGTACAAGTA | |  |
| Agtrl1_6 | CACCATCATGCTGACATGTTA | |  |
| Agtrl1_7 | ACCCTGAAACTTGAAGAGTAA | |  |
|  |  |  |  |
| **shRNA** |  |  |  |
| Gpr27 | gcgtggtgtgtttcctcttcaacttcaagagagttgaagaggaaacacaccacgctttttt | | |
